# Vegetarianism and Ill-Temper

**Published:** 1869-11

**Authors:** 


### VEGETARIANISM AND ILL-TEMPER.

SOLOMON was a great lover of beefsteak; and when he wanted a fit comparison with one of the meanest and lowest traits of our nature, to wit—a bad temper, he compares it to a vegetable dinner! saying that even a "dinner of herbs," with love and affection, was preferable to the most splendid table, marred with the presence of ill-nature. As writers of note are found, sooner or later, to have treated of things coming under their own experience and observation, we cannot resist the conclusion that Solomon had a "tartar" in his household. Other Solomons have the same. Steele had some experiences of a growling, grumbling ill-nature; and seems to have had a woman in his eye, when he declared: "A bad temper is a curse to the possessor. To hear one eternal round of complaint and murmuring; to have every pleasant thought scared away by this evil spirit, is a sore trial. It is like the sting of a scorpion, a perpetual nettle, destroying your peace, rendering life a burden. Its influence is deadly. The purest and sweetest atmosphere is converted into a deadly miasm, wherever this evil genius prevails. It is allied to martyrdom, to be obliged to live with one of a complaining temper. One string out of tune will destroy the music of an instrument, otherwise perfect. So, if all the members of a family do not cultivate a kind and affectionate temper, there will be discord, and every evil work."

Solomon and Steele evidently had their eye on the family relation. Oh, the curse, the living martyrdom of having one member in a household, whose low-bred nature, exhibiting itself in every hour of waking existence, clouds the brow of the parent, petrifies the glad smile of childhood, fixes a stern hatred on the heart of the servant, and fills the breast of the guest, or stranger, with sadness and inexpressible contempt!

In the estimation of the wisest of men, the distance between a dinner of beefsteak and vegetables was almost immeasurable; but, between vegetables, with lovingness, and a splendid repast, with a carping, grumbling nature, there could be no comparison, and he gladly chose the former. Let, then, the snarling curs, fortunately met with only here and there, make a note of this, if they can but know their picture; and remember, that to see no beauty in any flower, to feel no warmth in any sunshine, to draw no lovingness from any smile, is, of all temperaments, the most to be pitied, the worst to be feared.

